# A tailored indoor setup for reproducible passive daytime cooling characterization

**DOI:** 10.1016/j.xcrp.2022.100986

**Published:** 2022-08-17

**Authors:** Qimeng Song, Thomas Tran, Kai Herrmann, Tobias Lauster, Maximilian Breitenbach, Markus Retsch

**Affiliations:** 1 Department of Chemistry, Physical Chemistry I, University of Bayreuth, 95447 Bayreuth, Germany; 2 Bavarian Polymer Institute, Bayreuth Center for Colloids and Interfaces, and Bavarian Center for Battery Technology (BayBatt), University of Bayreuth, 95447 Bayreuth, Germany

**Keywords:** radiative heat transfer, sub-ambient cooling, photonic cooling, space heat sink, cooling power measurement

## Abstract

Passive daytime cooling materials can lower global energy consumption owing to their autonomous cooling capability. Although a significant number of passive cooling materials have been developed recently, their performance characterization is still challenging. Field tests experience high variability due to uncontrollable changes in environmental conditions. Here, we design an indoor setup to characterize the performance of passive cooling materials reproducibly and independently of weather and season. Outdoor measurement conditions are approximated using a liquid-nitrogen-cooled aluminum dome, a solar simulator, and a wavelength-selective inverse sky-window filter. In contrast to outdoor measurements, the results of various reference materials show remarkable precision and repeatability. Additionally, the impact of solar light intensity and temperature on the passive cooling performance can be experimentally investigated. Our setup is a first step in the development of a standardized test method to bring accuracy, reproducibility, and comparability to the emerging field of passive cooling materials.

## Highlights

• Powerful indoor setup for daytime passive cooling characterization
• High stability and reproducibility
• Control of solar intensity and ambient temperature
• Robust, cost-effective, and easy to build

## Graphical abstract

**Figure:**
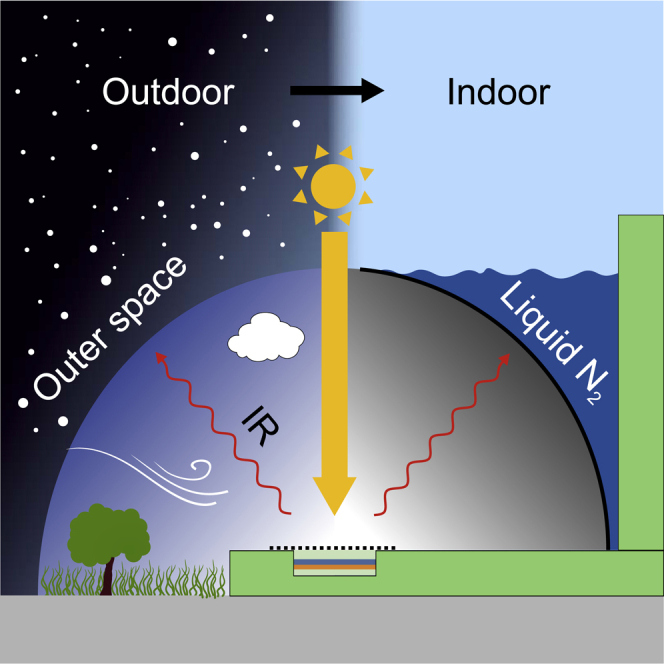

## Teaser

Standardized test methods are urgently required to characterize the performance of passive daytime cooling materials in a reliable and reproducible way worldwide. Song et al. introduce a robust test setup that unites cool space, solar radiance, and ambient environmental conditions in a compact indoor test setup with superior characterization performance.

## Introduction

Passive daytime cooling has emerged as a strong candidate to alleviate the global energy demand for cooling.^1^^,^^2^ It conveys heat from a material to outer space through the atmospheric window (8–13 μm) without external energy consumption. For an ideal daytime passive cooling performance, low absorption in the solar range (0.3–2.5 μm) and high emission over the mid-infrared (MIR) region is stringently required. In the last few years, advanced fabrication techniques have led to various novel materials, including photonically structured materials,^3^, ^4^, ^5^ hybrid composites,^6^, ^7^, ^8^, ^9^ highly porous materials,^10^, ^11^, ^12^, ^13^ and hierarchically structured materials.^14^, ^15^, ^16^, ^17^ These classes of materials promote the development of devices for daytime passive cooling applications.

Two essential techniques are usually used to evaluate a material’s passive cooling performance: optical spectroscopy and field testing.^2^^,^^18^ The former determines the spectral absorption of a material in both the solar and MIR regions. Utilizing a theoretical model based on energy balance considerations, the net passive cooling power of a material can be calculated. Li et al. introduced a simple figure of merit to fairly assess the performance of distinct cooling materials based on their optical properties.^19^ However, the comprehensive optical properties, including angle and temperature dependence, of a material, are rather hard to access, especially for complex materials, e.g., multilayer composites, self-adaptive metamaterials,^20^^,^^21^ and materials with irregular surface topography, making it difficult to achieve a precise comparison.

During field testing, the steady-state temperature of a sample and its cooling power at ambient temperature are obtained. However, outdoor measurements are impressionable and uncontrollable, and the outcome strongly depends on measurement conditions,^22^^,^^23^ e.g., geographical location, solar intensity, ambient temperature, humidity, wind speed, and air pressure. The uncontrollable and unsteady atmospheric conditions limit the comprehensive characterization of passive cooling materials firstly, and replication of the measurement results secondly. Due to this challenge, different materials cannot be compared reasonably. Therefore, a comparable and standardized test method is urgently required to push the development of passive daytime cooling materials forward. In contrast to outdoor environments, an indoor setup is independent of weather conditions and provides a stable and controllable environment for passive cooling characterization. A simple indoor setup for passive cooling characterization was reported by Zhou et al.^24^ By using liquid-nitrogen-cooled black aluminum foil as a heat sink, the nighttime passive cooling behavior of a PDMS film was imitated. However, their indoor setup did not include a light source. The characterization was thus limited to nighttime conditions. A similar setup was also constructed by Park et al. in a glovebox.^25^ With applying a solar simulator, the characterization can be performed in the presence of solar light. Very recently, a hybrid refrigerative thermoelectric cooling system was built by Wong et al. to simulate the radiative cooling effect artificially under controlled conditions.^26^ Their sophisticated setup achieved a reasonable accuracy (deviation of 17%–33%). Still, a repeatable and comprehensive characterization method for passive daytime cooling materials remains an enormous challenge.

In this work, we present a tailored indoor setup for comprehensively characterizing the performance of daytime passive cooling materials. The setup allows measurements with and without illumination of the sample, analogous to daytime and nighttime field testing, respectively. It consists of a liquid-nitrogen-cooled, hemispherical aluminum (Al) dome as a heat sink and an air mass (AM) 1.5 solar simulator as a light source. To the best of our knowledge, our method is the first indoor setup for the experimental characterization of passive cooling materials in both nighttime and daytime, with outstanding repeatability showcased for three distinct materials, namely, a silver (Ag) mirror, a polydimethylsiloxane (PDMS) film, and a graphite coating. Furthermore, our setup can experimentally determine the impact of environmental changes, such as the ambient temperature or solar irradiation intensity, on the material’s cooling performance. Such a parametric investigation is unfeasible with field tests due to uncontrollable environmental conditions. Lastly, our indoor setup is robust and simple to build, opening a promising pathway to quantitatively compare passive cooling materials designed in different research groups.

## Results and discussion

### Indoor setup design

The most important aspects of passive cooling field testing that a feasible indoor setup must capture are (1) radiative heat transfer from the sample to outer space; (2) illumination of the sample by the sun; and (3) measurement at moderate temperatures. The realization of these key measurement aspects can each be attributed to distinct parts of the proposed setup.

#### Radiative heat transfer

**Figure 1:**
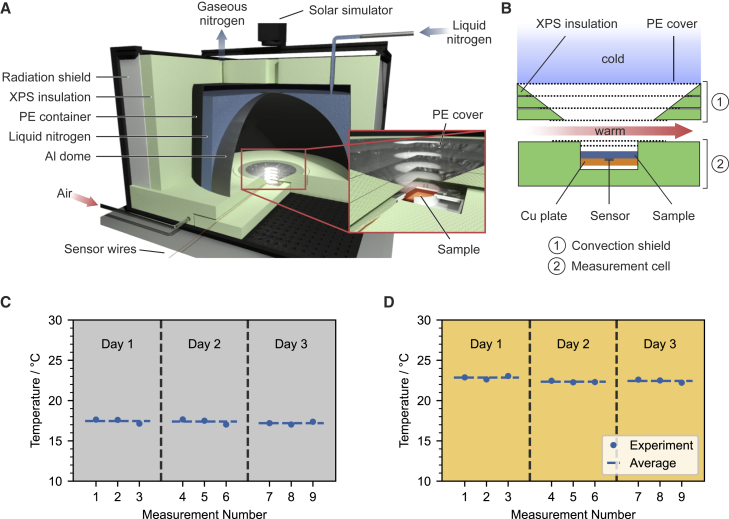
Indoor setup design and repeatability (A and B) Schematic of the indoor setup for characterizing passive daytime cooling. A liquid-nitrogen-cooled aluminum dome imitates outer space, while a solar simulator illuminates the sample. A convection shield in combination with XPS minimizes non-radiative heat transfer between the dome and sample. Detailed schematic of the sample holder (B). A warm gas flow between the convection shield and the sample holder allows controlling the temperature inside the measurement cell. (C and D) The steady-state temperature of an Ag mirror measured (C) w/o and (D) w/ solar light. Results show outstanding precision that cannot be achieved with outdoor measurements.

The samples emit hemispherically to outer space, which acts as a heat sink, with a temperature of ∼ 3 K. To imitate this behavior, we utilize a hemispherical Al dome with a diameter of 60 cm. The high thermal diffusivity of Al ensures a homogeneous temperature distribution across the entire surface. The inner surface of the dome was coated with graphite to enhance its broadband absorption, thus resembling space as a heat sink for radiative heat transport. A polyethylene (PE) container is imposed on the hemisphere, creating a reservoir around the Al dome. Liquid nitrogen is filled inside this reservoir and cools the dome down to ∼ 80 K. The entire setup is thermally insulated using extruded polystyrene foam (XPS, Styrodur, BASF) with a thickness of 8 cm to prevent cold loss. A schematic of the indoor setup is shown in Figure 1. Detailed dimensions and a photograph are in Figure S1. During the measurement, a small steady influx of liquid nitrogen compensates for all remaining heat losses to the environment. The sample is placed in a homemade measurement cell constructed by XPS under the center of the dome. Low-density polyethylene (LDPE) foils were applied above the sample to prevent convection.

#### Solar illumination

The average solar irradiation is generally presented by the AM 1.5 spectrum with a power of ∼ 1000 W m^−2^, which is well established in the characterization of photovoltaic devices.^18^ In the laboratory environment, a solar simulator was placed directly on the top of the dome to provide AM 1.5 solar light with an illumination area of 5 × 5 cm^2^. The hole in the dome accounts for 0.8% of the total area seen by the sample and is thus negligible. The similarity between the light of the solar simulator and the sun is shown in Figure S2. The light hits the sample at an angle of 8° to prevent back reflection into the solar simulator.

#### Sample temperature

The most challenging part of the indoor setup is to keep the sample at moderate temperatures while cooling the dome down to liquid nitrogen temperatures. The significant temperature difference across the short distance between the cooled dome and the sample holder naturally leads not only to the desired radiative heat transfer but also to undesired convection and conduction. Conduction is minimized by employing XPS insulation in combination with air gaps between the sample and the dome. Several LDPE foils (thickness ∼ 15 μm), possessing a high solar and IR transparency, act as a convection barrier between the sample and the cold air inside the dome. These PE layers reduce the solar radiation intensity in the sample by 25%, due to absorption, reflection, and scattering. Furthermore, the reduction of the transmittance is angle-dependent (Figure S3). Previous reports showed that angles <60° have a dominant contribution to the emission of passive cooling materials.^4^^,^^27^^,^^28^ Therefore, the convection shield made from XPS and LDPE has a cone-shaped inner part, with a zenith angle of 60° to reduce the thermal loss (conduction and convection) area while marginally blocking the pathway of thermal radiation from the sample. Conductive heat transport from each gas compartment to the next would eventually cool the air below the convection shield. A heated flow of dry air offsets this effect. The constant airflow and the stable laboratory environment (Figure S4) ensure highly stable and reproducible experiments. Moreover, controlling the temperature by tuning the airflow temperature allows for experimentally setting the ambient temperature and elaborating the temperature-dependent performance of passive cooling materials.

### Performance assessment

We investigated the repeatability of the indoor setup without (w/o) and with (w/) solar light. Multiple measurements of the steady-state temperature of an Ag mirror on different days are shown in Figures 1C and 1D. An SD of ± 0.24 K and ± 0.26 K was obtained from nine measurements over three consecutive days w/o and w/ light, respectively. This minor deviation shows that our indoor setup possesses outstanding repeatability in both nighttime and daytime-like measurements. When shining solar light with one sun power on the Ag mirror, the temperature increased by about 6 K. Since a LDPE foil is applied to the measurement cell to prevent convection, we thus attribute the temperature increase to the greenhouse effect caused by the parasitic solar light absorption of the Ag mirror and sample holder. This phenomenon has also been observed in outdoor measurements.^29^^,^^30^

**Figure 2:**
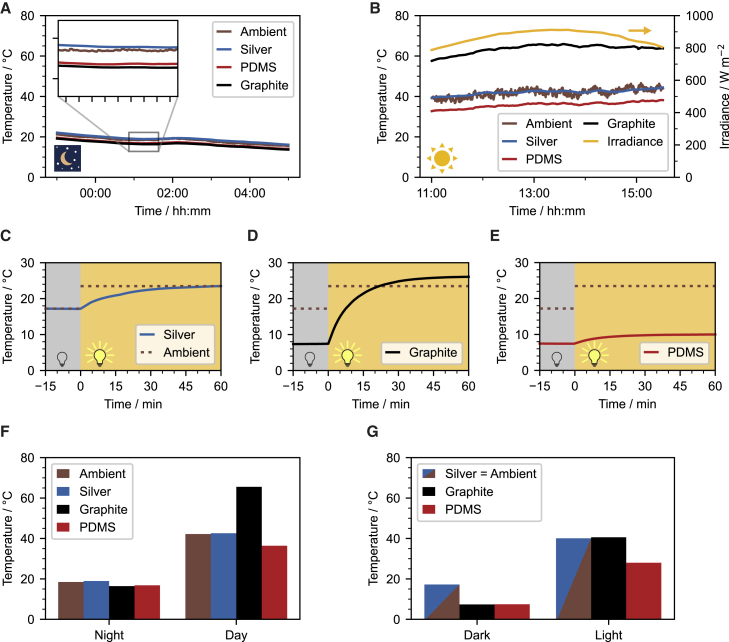
Performance assessment (A and B) Outdoor rooftop measurement for the reference materials at (A) nighttime and (B) daytime. The measurement was carried out under a clear sky, on June 17–18, 2021 in Bayreuth, Germany. (C–E) Indoor measurements of the (C) Ag mirror, (D) PDMS film, and (E) graphite coating. The turn-on point of the solar simulator was defined as 0 min. The dashed lines indicate the respective ambient temperatures for measurements w/o and w/ light. (F and G) The steady-state temperature of the reference materials in the (F) outdoor and (G) indoor measurements, respectively.

For outdoor measurements, an intuitive definition of the ambient temperature is the air temperature, which can be measured simultaneously with the sample temperature. For indoor measurements, however, the environment temperature results from the interplay between the cold dome and the warm gas flow. The large temperature gradient between the sample holder and the dome surface prevents a meaningful ambient temperature measurement in the vicinity of the actual sample. Therefore, an alternative way to define the ambient temperature is needed. Ag possesses a low emissivity in both the solar and IR region and, therefore, closely resembles the ambient temperature inside of the measurement cell without any major radiative heat losses or gains. We confirm this assumption by comparing the Ag mirror temperature with the ambient temperature in a field test (Figures 2A and 2B), where both temperatures almost overlap. The ambient temperature for the indoor setup is thus defined as the temperature obtained with an Ag mirror.

To verify the reliability of the indoor setup, we analyzed three reference materials: an Ag mirror, a PDMS film on an Ag mirror, and a graphite-coated silicon wafer. The chosen materials possess very different optical properties. Ag has a very low emissivity in both the visible and MIR regions, as outlined above. Furthermore, it has been widely applied to the backside of daytime passive cooling devices to minimize solar absorption.^6^^,^^31^ PDMS is a good passive cooling material due to its inherent selective emissivity in the MIR region. By combining the PDMS film and an Ag mirror, a practical daytime passive cooler is easily constructed.^24^^,^^32^ As the last sample, we use a graphite coating exhibiting broadband absorption in the solar and MIR regions. The spectral emissivity of all reference materials is shown in Figure S5.

Measurements of the reference materials were carried out w/o and w/ light irradiation (Figures 2C–2E). For measurements w/o light, a steady-state temperature of 17.2°C, 7.5°C, and 7.4°C was observed for the Ag mirror, the PDMS film, and the graphite coating, respectively. The PDMS film and graphite coating show a steady-state temperature ∼10 K lower than the Ag mirror. This is caused by the emissivity dependence of the radiative heat transfer. In contrast to the PDMS film and graphite coating, the Ag mirror shows negligible emissivity in the MIR region.

Subsequently, the solar simulator was used to simulate daytime measurements. After the reference materials reached a steady-state temperature in the dark, the solar simulator with one sun power was switched on. The temperature of all reference samples increased, and a new steady-state temperature was reached within 1 h. Compared with the Ag mirror (+6.3 K) and the PDMS film (+2.6 K), the temperature increase of the graphite coating was substantial (+18.8 K). The temperature increase of the Ag mirror can be explained by the absorption of the sample holder and the greenhouse effect. The different light response of the reference materials occurs because graphite has a significantly higher absorption in the solar regime. In addition, more than a 10 K difference in the steady-state temperature was observed between the Ag mirror and the PDMS film. The much lower final temperature of PDMS film demonstrates the good passive daytime cooling performance of the PDMS film agreeing with field testing in the literature.^24^^,^^29^

To compare the results of the indoor experiment with the conventionally used field testing, outdoor measurements of the reference materials (Figures 2A and 2B) were conducted in both nighttime (23:00–05:00) and daytime (11:00–15:30). The setup for the outdoor measurements is shown in Figure S6. The PDMS film and graphite coating show similar steady-state temperatures at nighttime, ∼2.5 K lower than the Ag mirror (Figure 2F). During the daytime, the Ag back mirror of the PDMS sample reflected most solar irradiation, while the PDMS transferred heat to outer space via IR radiation. Therefore, the PDMS film keeps its sub-ambient temperature even with an average solar intensity of around 850 W m^−2^. In contrast, the graphite coating absorbed considerable solar energy and warmed up to ∼60°C.

Indoor measurements were performed with the respective ambient outdoor temperatures, 17°C and 40°C (Figure 2G). We observe that the absolute values of steady-state temperature for the reference materials obtained from indoor and outdoor measurements do not agree with each other. A daytime sub-ambient cooling by 6.2 K was observed from an outdoor measurement of a PDMS film, while 12.1 K was obtained from the indoor setup. We attribute this difference to two main contributions. First, the black-coated Al dome represents a broadband black body. More radiative heat can be transferred to it compared with the higher selectivity of the atmospheric window in outdoor measurements. Hence, the sub-ambient cooling power is increased. Second, the irradiance in the outdoor measurement is 900 W m^−2^, while the irradiance in the indoor measurement is only 750 W m^−2^. Consequently, the absorbance in the visible spectrum leads to a higher heat input for the outdoor case. Considering these effects, the values agree reasonably well. The reproducible values of the indoor setup allow for better comparison between different measurements as displayed in the literature. PDMS samples measured at different locations and times show an even larger range of temperature reductions. For instance, Zhou et al. observed a sub-ambient cooling of 11 K at Buffalo, NY, USA (February 2018).^24^ Zhu et al. reported 3.3 K at Nanjing, China (November 2019),^33^ and our previous study showed 7.4 K at Bayreuth, Germany (April 2020).^29^ The disparity in the investigation of similar PDMS films from different groups is attributed to the distinct measurement conditions, e.g., ambient air temperature, humidity, and solar irradiation. Even subsequent measurements with the same setup from one group at a fixed location are prone to fluctuations, due to the equilibration time and the natural changes in temperature, humidity, and solar radiation. Consequently, multiple tests are typically run simultaneously to allow for comparability among different samples. In contrast, the proposed indoor setup enables measurements with a predetermined condition and, thus, allows a quantitative comparison of measurements from different days.

### Cooling power characterization

**Figure 3:**
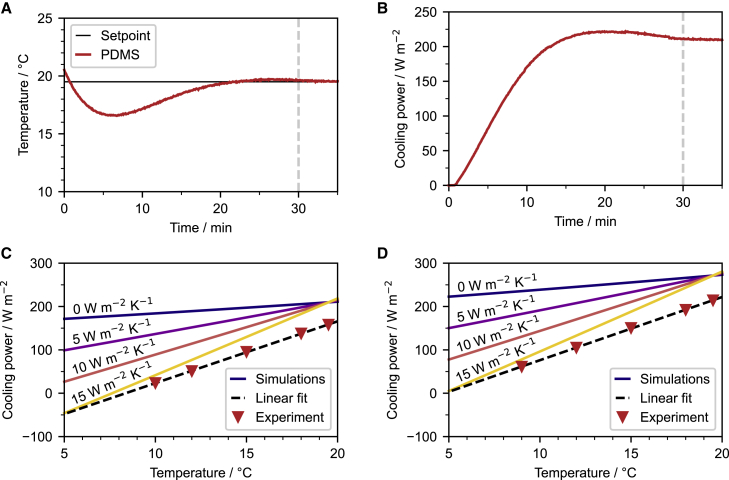
Comparison with numerical calculations (A and B) Temperature tracking (A) and net cooling power measurement (B) of a PDMS film (∼88.4 μm) via the indoor setup. (C and D) A steady net cooling power could be obtained within 30 min. Numerically calculated net cooling power as a function of emitter temperature with different non-radiative heat transfer coefficients (*h*_c_) for (C) a thin PDMS film (∼19.2 μm) and (D) a thick PDMS film (∼88.4 μm), based on the configuration of the indoor setup. The respective experimental values are measured and plotted to estimate *h*_c_ for our setup. Values of 11.6 and 11.2 W m^−2^ K^−1^ were obtained for the thin and thick PDMS samples, respectively. For the experiments, the setpoint was 19.5°C and solar light was excluded.

Besides the sub-ambient temperature that a passive cooling material can reach, its net cooling power is another important parameter for quantifying the cooling performance. The standard technique to measure the net cooling power is using a feedback-controlled electrical heater underneath the sample to maintain the ambient temperature. As a result, the recorded input heating power is equivalent to the net cooling power.^4^ However, the cooling power obtained from outdoor measurements, in most cases, fluctuates over time due to the unstable conditions, mainly solar intensity, wind speed, and cloud coverage. Hence, such outdoor measurements are typically carried out for many hours and merely reach a temporary steady-state condition, making quantification of the cooling power difficult.^7^^,^^11^^,^^34^ By contrast, a steady net cooling power can be measured with the presented indoor setup in a matter of minutes. The ambient temperature is set to the Ag mirror temperature determined under the same measurement condition. We assume that at this temperature, no non-radiative losses, i.e., convection and conduction, occur. As shown in Figures 3A and 3B, the net cooling power of a PDMS film (∼ 88.4 μm) was obtained within 30 min. The PDMS film exhibits a net cooling power of about 200 W m^−2^ at an ambient temperature of 19.5°C, w/o solar light. Compared with the net cooling power of the PDMS-based passive cooling devices reported in the literature, i.e., up to ∼130 W m^−2^,^31^ the value obtained from the indoor setup is relatively high. This is attributed to the absence of atmospheric thermal radiation outside of the sky-window range in the indoor setup.^35^ Nevertheless, characterizing the passive cooling power with the indoor setup allows for a precise and defined measurement with a reasonably fast equilibration time.

For materials exposed to the clear sky, the net cooling power can be calculated with the radiative model, *P*_cool_ = *P*_mat_ − *P*_sun_ − *P*_atm_ − *P*_nonrad_. Here, *P*_mat_ is the thermal irradiation power of the emitter, *P*_sun_ is the solar power absorbed by the material, *P*_atm_ is the absorbed power from the atmosphere, and *P*_nonrad_ is the absorbed power due to conduction and convection. *P*_nonrad_ can be expressed as *P*_nonrad_ = *h*_c_ · (*T*_atm_ − *T*_mat_), where *h*_c_ is the non-radiative heat transfer coefficient. For the indoor setup, the inner space is filled with nitrogen and the path between the sample and the dome is short. Thus, *P*_atm_ can be omitted and the equation can be simplified to *P*_cool_ = *P*_mat_ − *P*_sun_ − *P*_nonrad_ and *P*_cool_ = *P*_mat_ − *P*_nonrad_ for daytime and nighttime, respectively. It needs to be noted that *h*_c_ varies from measurement to measurement, because of the distinct measurement conditions, and the estimation of *h*_c_ has been widely conducted,^4^^,^^36^ whereas, it should not depend on the material optical properties.

To better understand our indoor setup and verify that *h*_c_ is indeed independent of the optical properties for all indoor setup measurements, we calculated the theoretical net cooling powers of PDMS films with various optical properties and compared them with the actual indoor measurements. The *h*_c_-dependent net cooling power of PDMS films with two different thicknesses, 88.4 and 19.2 μm, was calculated via the radiative cooling model based on the indoor setup configuration. The model was thoroughly discussed in our previous work, which demonstrated the thickness dependence on optical properties and passive cooling performance.^29^ The complex refractive index of PDMS films used in the calculation was obtained from the literature.^37^^,^^38^ To determine *h*_c_ of our indoor setup measurements, the net cooling powers of the PDMS samples were measured at five different temperatures. We observed a linear relationship between the sample temperature and the cooling power that agreed with the expected trend obtained from the numerical calculations (Figures 3C and 3D). Based on the linear fitting of the measurement points, we estimate *h*_c_ of the indoor measurements to be 11.6 and 11.2 W m^−2^ K^−1^ for the measurements of thin and thick PDMS films, respectively (Figure S7). The consistent *h_c_* value obtained from the indoor measurements with different samples proves the stability and reliability of the indoor setup. In addition, we observed a slight offset between the measured and calculated net cooling power. We attribute this to the approximations in the theoretical model, the adopted complex refractive index value from the literature,^29^ and the uncertainty of the measurement.

### Variation of environmental parameters

**Figure 4:**
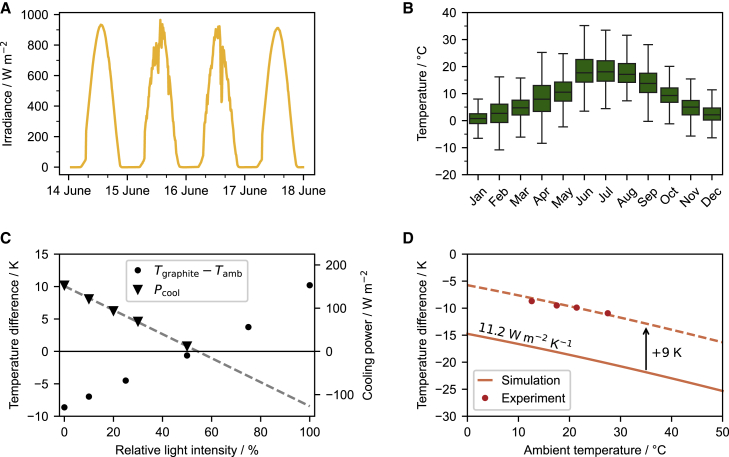
Variation of environmental parameters (A) Solar irradiance over four days in Bayreuth, Germany. (B) Ambient air temperature of different months in Bayreuth, Germany, over the last three years. The error bars represent the standard deviation of the data. (C) Impact of solar intensity on the passive cooling performance of a graphite coating. The temperature difference (*T*_graphite_ − *T*_amb_) and net cooling power (*P*_cool_) show a linear trend when increasing the solar light intensity from 0% to 100% of one sun (∼1000 W m^−2^). (D) The impact of ambient temperature (*T*_amb_) on the cooling performance of a PDMS film. Both the simulation and experiment show that *T*_amb_ enhances the cooling performance leading to a decrease in the temperature difference (*T*_PDMS_ – *T*_amb_).

Solar irradiance and ambient temperature vary with time and location. They strongly influence the cooling capacity of passive daytime cooling devices. As shown in Figures 4A and 4B, solar irradiance changes from 0 to ∼950 W m^−2^ during a summer day and ambient air temperature changes between about −10°C to about 35°C over a year in Bayreuth, Germany. How do temperature and solar radiance influence the cooling performance? In our indoor setup, the solar irradiance can be changed between 0 and 100% of one sun via the solar simulator. We, therefore, examined the sub-ambient cooling as well as the net cooling power of a graphite coating at various solar intensities, from 0% to 100%. As illustrated in Figure 4C, the temperature difference (*T*_graphite_ − *T*_amb_) increased from about −10 K to +10 K with increasing solar intensity from 0% to 100%. Concomitantly, the net cooling power declined gradually. Moreover, the temperature difference and the net cooling power showed a linear trend with respect to the solar intensity.

The temperature controllable gas flow allows tuning of the ambient temperature in the measurement cell between 10°C and 35°C (Figure S8). The temperature dependence of blackbody radiation is well known. An increasing emitter temperature enhances the thermal irradiation, and, therefore, the cooling performance. We measured the steady-state temperature of a PDMS film (88.4 μm) at various ambient temperatures. We found that with increasing the ambient temperature, the cooling performance was also enhanced, as indicated by the increased sub-ambient cooling (Figure 4D). The sub-ambient cooling rose from 8.7 K to 11.0 K, with increasing the ambient temperature from 12.6°C to 27.5°C. We confirmed this temperature dependence by a numerical calculation with *h*_c_ = 11.2 W m^−2^ K^−1^. Despite the offset between the experimental and theoretical values (∼9 K), the trends agree well.

In general, passive cooling materials can be divided into two groups. The first group is selective emitters, which emit only at the atmospheric window (8–13 μm). These emitters transfer heat directly to outer space without interference from the atmosphere. The second one is broadband emitters. Broadband emitters emit not only at the atmospheric window but also outside, resulting in heat exchange with the atmosphere itself.^39^ It is controversial which kind of emitter is better suited for passive cooling applications.^31^^,^^40^ In its current form, our setup is best suited to compare emitters of the same type, i.e., compare broadband with broadband and selective with selective emitters. However, the discrimination between broadband and selective emitters is limited due to the lack of an atmospheric window. Directly simulating the atmosphere within the indoor setup is rather challenging.

**Figure 5:**
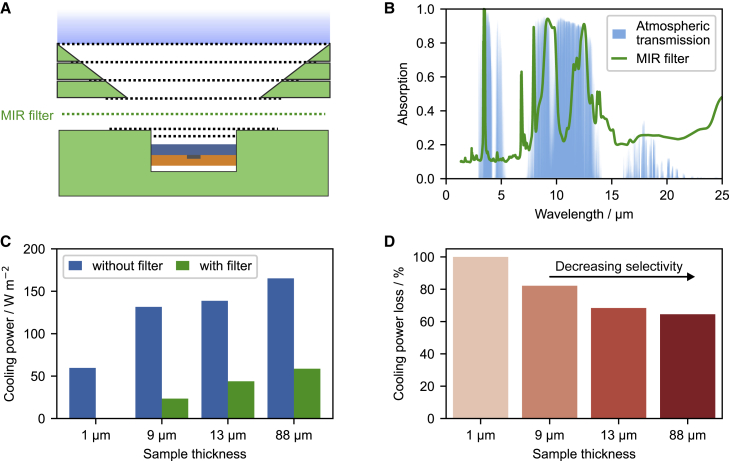
Distinguishing broadband and selective emitters (A) An MIR filter consisting of PDMS on PE is inserted between the sample and the convection shield. (B) The absorption spectrum of the PDMS-PE filter is comparable with the transmission of the atmosphere. (C) Cooling power measurements of differently thick PDMS samples with and without the filter. With increasing thickness, the cooling power rises. The introduction of the MIR filter results in a significant reduction of cooling power. (D) The relative cooling power loss decreases with increasing PDMS film thickness. The loss correlates with the emission selectivity of the samples.

Instead of equipping the indoor setup with a direct MIR filter with a transmission similar to the atmosphere to further imitate the field testing, an inverse MIR filter that emits only in the atmosphere window regime can be introduced. By placing the inverse MIR filter, which possesses ambient temperature, between the sample and the cold dome during the measurement, only the spectral radiation outside the atmosphere window regime can reach the cold dome (Figure 5A). The filter will block the rest. Selective emitters can thus be distinguished from broadband emitters by comparing the proportion of cooling power loss in a two-step measurement without and with the inverse MIR filter installed. The radiation in the atmosphere window regime contributes distinctly to the radiative cooling ability of broadband emitters and selective emitters.

We demonstrate such an inverse MIR filter by coating an LDPE foil with a thin layer of PDMS. A thin PDMS film with the desired thickness on an LDPE foil ensures a high absorption in the regime of 8–13 μm and simultaneously high transmission in the remaining spectral range (Figure 5B). The cooling power of PDMS films with different thicknesses, i.e., 712 nm and 8.6, 13.1, and 88.4 μm, is determined w/o and w/ the PDMS-PE filter to prove the concept (Figures 5C and S9). The optical properties of the PDMS films show that with increasing film thickness, they gradually transition from a selective to a broadband emitter (Figure S10). Consequently, the cooling power without the MIR filter increased gradually. This is in good agreement with our previous work based on outdoor measurements.^29^ The cooling power reduces dramatically when applying the PDMS-PE filter. The decrease varies for the differently thick PDMS films. To highlight the cooling power difference caused by the PDMS-PE filter, the relative power loss was calculated as power loss = (*P*_w/o_ − *P*_w/_)/*P*_w/o_ and is shown in Figure 5D. The increasing thickness of the PDMS films reduces the cooling power loss, which indicates that the PDMS-PE filter affects selective emitters more importantly than broadband emitters. Besides, we also calculated the cooling power loss of the PDMS films w/and w/o atmosphere with outdoor conditions (Figure S11). Despite the deviation of the absolute value between the experiment and the simulation, the trend agrees well. The deviation is mainly attributed to the imperfect match between the PDMS-PE filter and the atmosphere. The inverse MIR filter design can assess the influence of the sky-window transmission on the cooling power but is not suitable to compare the temperature reduction with outdoor measurements. A direct temperature comparison required a filter matching the sky-window properties.

Although it is hard to imitate the atmosphere in the indoor setup, with the inverse MIR filter, the indoor setup can distinguish between selective and broadband emitters. The possibility to add a tailor-made filter to our setup further expands the scope for its applicability to characterize passive daytime cooling materials close to field testing conditions. Further additions may include filters that account for a specific relative humidity or cloud coverage. Another class of filters may address the angular dependence of the thermal emission of a given material by controlling the view factor of the dome in dependence on the polar angle. Such a measurement capability is highly demanded to characterize emissive materials with diffusive reflectance properties. For instance, the heat management properties of smart textiles can be investigated with such a sample-dome layout, when operated at body heat and room temperature, respectively.^41^

In summary, we constructed a versatile indoor setup to thoroughly characterize the performance of passive cooling materials for both daytime and nighttime. Our setup combines a liquid-nitrogen-cooled Al dome with a solar simulator. Unlike conventional outdoor measurements, our setup allows controlling the measurement conditions, leading to outstanding reproducibility and time-saving measurements. Characterizing materials in a laboratory environment makes measuring and comparing materials independent of weather, time, and location. Additionally, the impact of solar intensity and ambient temperature on the cooling performance can be practically studied. Such comprehensive investigations are impossible for outdoor measurements due to the uncontrollable atmospheric conditions. We are convinced that our test setup is a first step toward a standardized passive cooling test routine. A standardized method to practically compare the cooling performance of various innovative materials from research groups all over the world is, however, a gatekeeper to turning passive cooling into a widespread and applied technology.

## Experimental procedures

### Resource availability

#### Lead contact

Further information and requests for resources should be directed to and will be fulfilled by the lead contact, Markus Retsch (retsch@uni-bayreuth.de).

#### Materials availability

This study did not generate new unique reagents.

### Preparation of reference materials

#### Ag mirrors

Ag with a thickness of 100 nm was thermally evaporated on a silicon wafer (r = 2.5 cm), followed by a deposition of 10 nm silicon oxide (SiO_2_) with a sputter coating step.

#### PDMS films

PDMS films with different thicknesses (88.4, 13.1, and 8.6 μm and 712 nm) were prepared on top of the Ag mirrors. For this, a prepolymer of PDMS (Sylgard 184, Dow Chemical) was mixed with a curing agent in a ratio of 10:1 (by weight) and degassed in a desiccator under vacuum. Subsequently, films with a thickness of 88.4 and 13.1 μm were prepared via spin-coating (1,000 and 3,000 rpm) on the Ag mirror. For films with a thickness of 8.6 and 13.1 μm, the prepolymer/cross-linker mixture was diluted to 75 wt % and 25 wt % solutions, respectively, with n-hexane. The films were then prepared via spin coating (3,000 and 4,000 rpm) on the Ag mirror. The PDMS layers were cured at room temperature for 48 h.

#### Graphite coating

The graphite coating is prepared by spray coating graphite (Cramolin, ITW Spraytec, Germany) onto a precleaned silicon wafer (r = 2.5 cm), followed by evaporation of the solvent at ambient temperature.

The layer thickness of the reference samples, namely PDMS films and graphite, was determined by using a three-dimensional (3D) laser scanning microscope (LEXT OLS5000, Olympus). A thickness of 88.4 μm, 13.1 μm, 8.6 μm, 712 nm, and 3.2 μm was obtained for the four PDMS films and the graphite coating, respectively.

#### PDMS-PE window

A prepolymer of PDMS (Sylgard 184, Dow Chemical) was mixed with a curing agent in a ratio of 10:1 (by weight) and degassed in a desiccator under a vacuum. The resulting mixture was diluted to a 50 wt % solution with n-hexane. A PDMS thin film was spin-coated (3,500 rpm) on an LDPE foil (thickness of around 15 μm), which was evenly attached to a silicon wafer (r = 7.5 cm). A PDMS-PE window was obtained by detaching the PDME-PE foil from the silicon wafer.

### Optical characterization with UV-Vis and FTIR spectroscopy

Broadband optical properties of the reference materials were characterized by ultraviolet-visible (UV-Vis) and Fourier transform infrared (FTIR) spectroscopy. UV-Vis reflectance of the reference materials was measured with a UV-Vis spectrometer (Cary 5000, Agilent Technologies) equipped with an integrating sphere accessory (Labspheres). A Spectralon diffuse reflectance standard (Labspheres) was used as a reference. The FTIR spectroscopy measurements were carried out on an IR spectrometer (Vertex 70, Bruker) coupled with a gold-coated integrating sphere accessory (A562, Bruker). A gold mirror was used as a reference. The absorptance (emittance) was calculated as absorptance (emittance) = 1 − reflectance. We assume that the transmission can be neglected because of the Ag layer at the back.

### Transmittance characterization of the convection shield

We used a pyroelectric sensor (FieldMaxII, Coherent) to measure the power of the solar simulator irradiation. Triplicate measurements were performed with and without the convection shield between the simulator and the sensor. The transmittance was calculated as 1 − *P*_w/ shield_/*P*_w/o shield_.

### Indoor measurements

Daytime measurements are imitated by applying solar light provided by a solar simulator (AX-LAN400, Sciencetech, Canada) with an illumination area of 5 × 5 cm^2^. For nighttime measurements, the solar simulator was turned off. For all indoor measurements, dried air was warmed up by a water bath with a controlled temperature and flushed the area between the convection shield and measurement cell. Liquid nitrogen was filled into the setup to cool down the Al dome. Before filling the liquid nitrogen into the setup, the inner space of the dome is flushed with N_2_ to remove air. Thus, no pronounced water condensation was observed on the convection shield. The temperature of the dome is maintained during the entire measurement by continuously filling liquid nitrogen into the setup. The sample temperature is measured with a thermocouple (type T) and collected by a digital multimeter (DAQ6510, Tektronix, Germany) every 5 s. To determine the steady-state temperature, data from the last 5 min of the measurement were averaged.

For the indoor measurements with the PDMS-PE window, the prepared PDMS-PE foil was placed above the sample holder at a distance of around 5 mm.

#### Repeatability test

An Ag mirror was used to check the repeatability of the setup. The temperature of the airflow was controlled by setting the water bath to 40°C. The steady-state temperature of the Ag mirror was measured three times per day on three different days, w/ and w/o one sun power of solar light (∼1000 W m^−2^).

#### Solar intensity dependence test

The graphite coating was measured to highlight the influence of solar intensity on the cooling performance. The temperature of the airflow was set to 40°C. The intensity of the solar light was varied from 0% to 100% of one sun power (∼1000 W m^−2^). The steady-state temperature and the cooling power of the graphite coating were then obtained under each condition. The net cooling power was measured by actively heating the graphite coating to keep it at the same temperature as an Ag mirror under the same conditions (water bath temperature and solar irradiance).

#### Ambient temperature dependence test

A PDMS film (88.4 μm) was applied to prove the temperature dependence of the thermal irradiation. The temperature of the airflow was set to 35°C, 40°C, 50°C, and 60°C. Subsequently, the steady-state temperature of the Ag mirror and the PDMS film was measured w/o the solar light.

### Rooftop measurements

Rooftop measurements for daytime and nighttime were carried out on the roof of a four-floor building (June 17–18, 2021, University of Bayreuth, Bayreuth, Germany) under a clear sky. The reference samples were each placed in identical homemade sample holders. The holders were thermally insulated by Styrofoam and covered with Mylar Al foil. Convective heat transfer was prevented by applying an LDPE foil, with a thickness of approximately 15 μm. The emitter temperatures were measured by Pt100 temperature sensors and recorded with a digital multimeter (DAQ6510, Tektronix, Germany) every 5 s. Temperatures between 1:00–1:30 and 13:00–13:30 were averaged to obtain steady-state temperatures. One sample holder covered with Al foil but without LDPE foil was used to obtain the ambient temperature. The solar irradiance data were collected from the weather station at the University of Bayreuth (Ecological-Botanical Garden, 400 m away from the rooftop measurement).

### Numerical calculations

The numerical calculations for theoretically estimating the steady-state temperature and cooling power of the PDMS films are based on a model that is described in our previous study.^29^ The broadband optical properties of the PDMS film are obtained from the literature.^37^^,^^38^^,^^42^ The sample was tilted 8° to avoid direct reflection of the solar light. For simplicity, this has been neglected in numerical calculations. A polar angle θ of 60° and an azimuthal angle of 360° was applied to the calculation based on the configuration of the indoor setup. We assume that no thermal radiation was emitted by the liquid-nitrogen-cooled dome.

To calculate the cooling powers of PDMS films with different thicknesses, i.e., 88.4, 13.1, 8.6 μm and 712 nm, with the outdoor condition, a polar angle θ of 90° and an azimuthal angle of 360° were applied. The ambient temperature of the emitter is set to 18.5°C, which is the preset temperature of PDMS films for indoor cooling power measurement. For emitters with a temperature as same as ambient temperature, the nighttime cooling power is calculated as *P*_mat_ − *P*_atm__._ *P*_mat_ is the total power emitted by the sample. *P*_atm_ is the absorbed power from the atmosphere. The cooling power loss is calculated as (*P*_mat_ − *P*_atm_)/*P*_mat_.

## Data Availability

All data from this study are available from the corresponding author upon reasonable request.

## Supplemental information

Document S1. Figures S1–S11

Document S2. Article plus supplemental information
