# Supplementary material for: A tailored indoor setup for reproducible passive daytime cooling characterization
Source: Cell Rep Phys Sci. 2022 Aug 17;3(8):100986. doi: 10.1016/j.xcrp.2022.100986 (PMC9391927; doi:10.1016/j.xcrp.2022.100986)
Supplement: Document S1. Figures S1–S11 [file mmc1.pdf]

Cell Reports Physical Science, Volume *3*

Supplemental information

A tailored indoor setup for reproducible passive daytime cooling characterization

Qimeng Song, Thomas Tran, Kai Herrmann, Tobias Lauster, Maximilian Breitenbach, and Markus Retsch

Supplemental Information

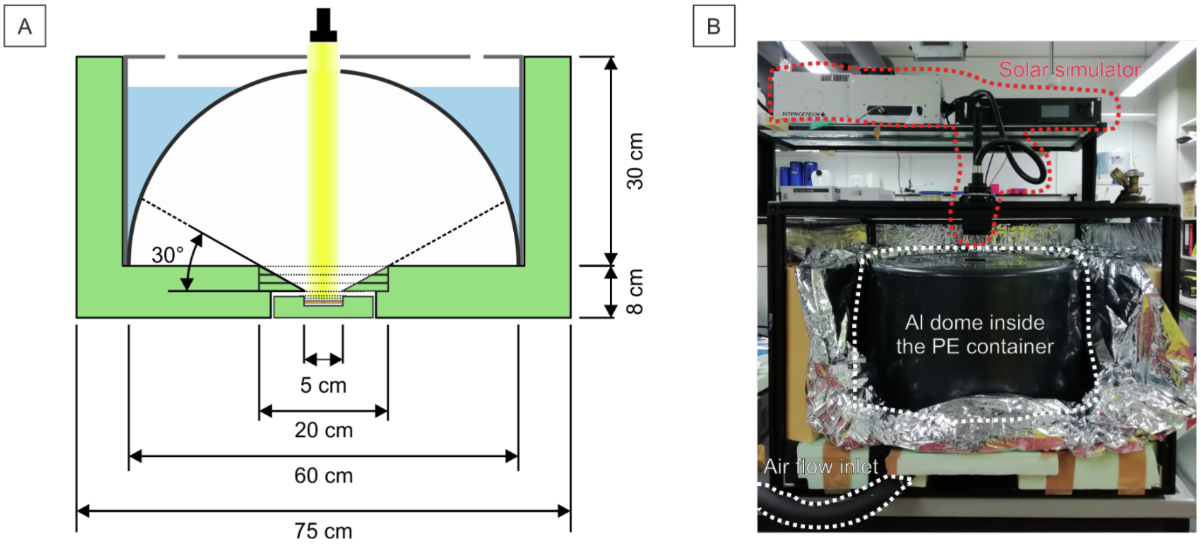

Figure S1. Indoor setup design. (A) Detailed dimensions and (B) photograph of the indoor setup. Related to Figure 1 and Indoor Setup Design.

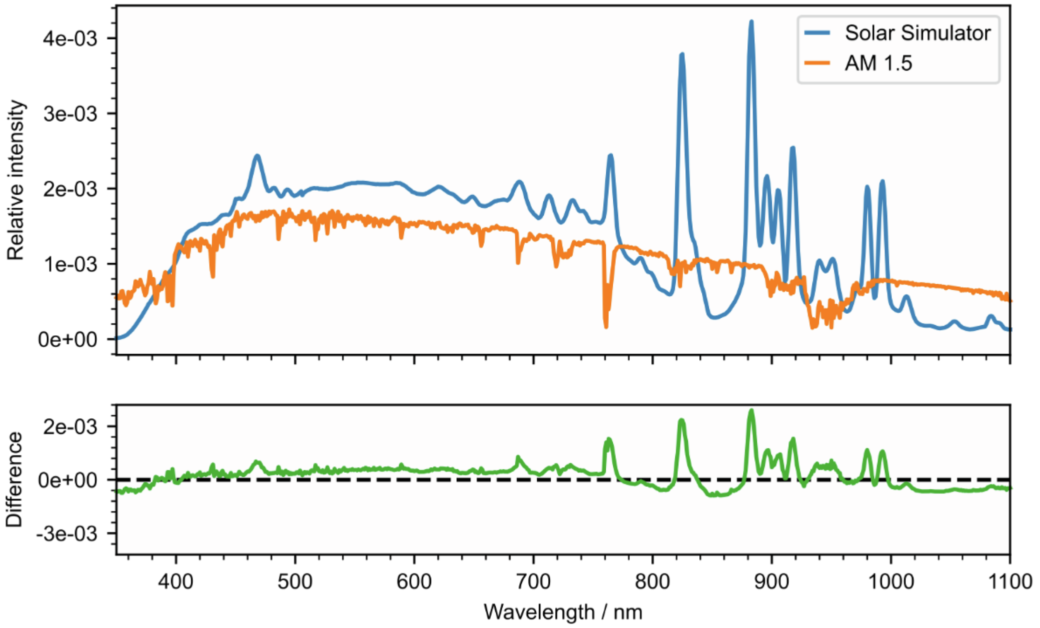

Figure S2. Comparison between the solar light from the sun (AM 1.5) and the solar simulator. The intensity shown is relative to the total intensity of the light source, i.e., the integral from 0 to infinity is 1 for each individual curve. Related to Indoor Setup Design.

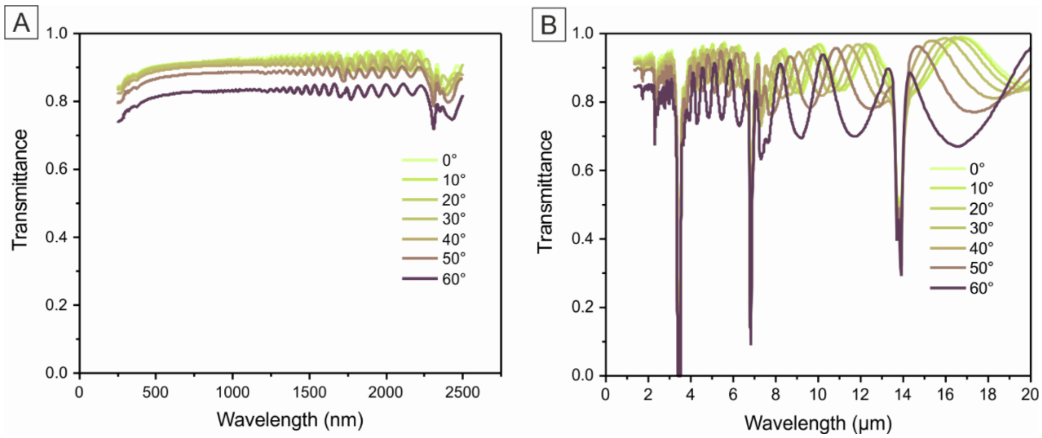

Figure S3. Angle-dependent optical properties in (A) UV/Vis and (B) IR regime for a single, 15 μm thick PE window used in the convection shield. Related to Indoor Setup Design.

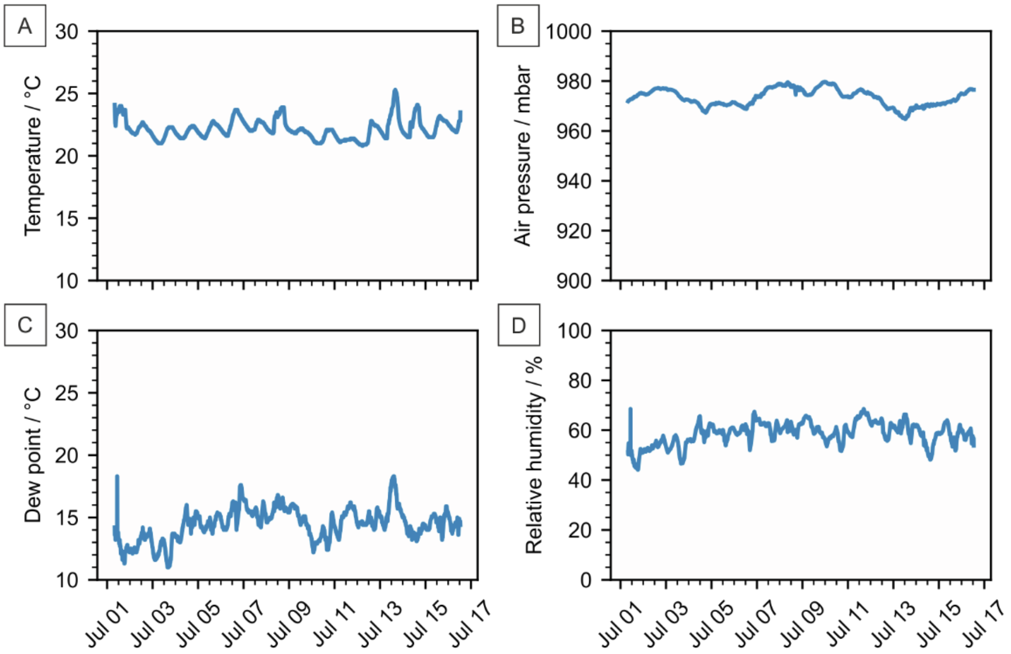

Figure S4. Laboratory conditions. (A) Ambient temperature, (B) air pressure, (C) dew point, and (D) relative humidity in the laboratory over a week. Related to Indoor Setup Design.

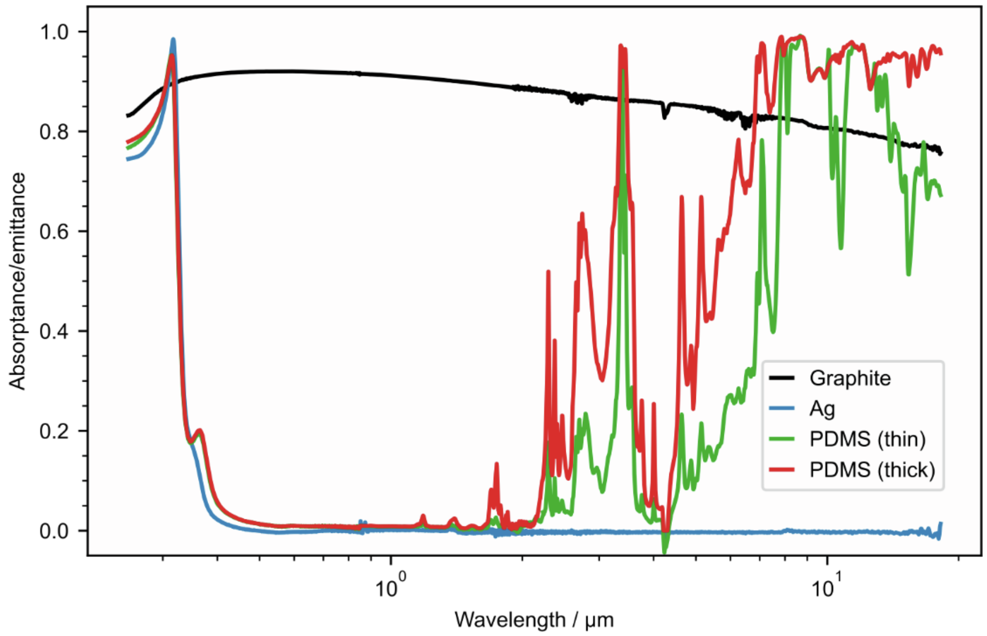

Figure S5. Optical properties of the reference materials used in this work, i.e., PDMS, Ag, and graphite, in the UV/Vis and MIR region. Related to Figure 2 and Performance Assessment.

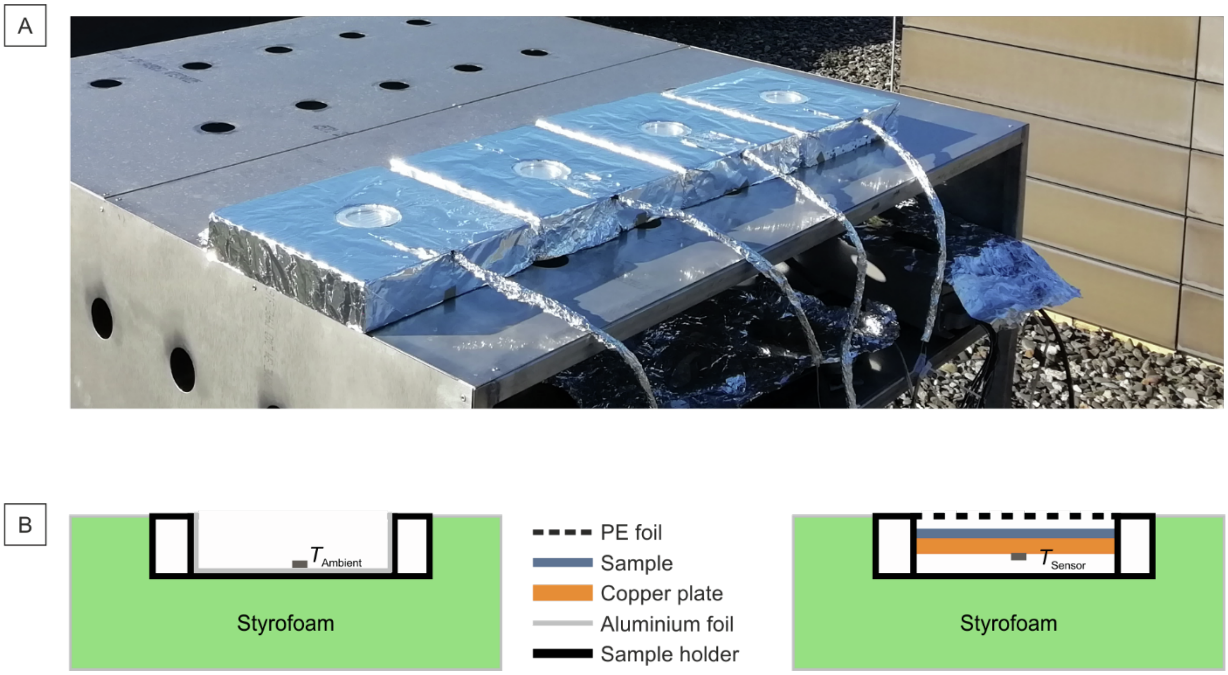

Figure S6. Outdoor setup design. (A) Photograph and (B) schematic of the setup for outdoor measurements. Related to Figure 2 and Performance Assessment.

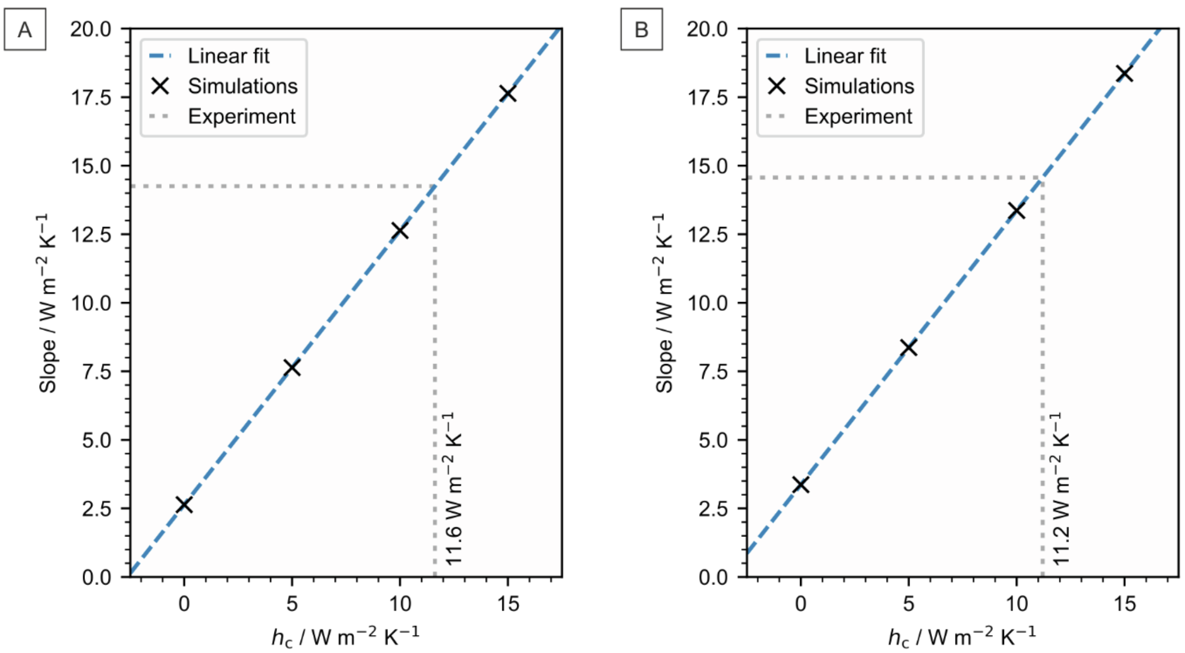

Figure S7. Determination of h_c for the thin (A) and thick (B) PDMS samples in the indoor setup measurement. The linear fit shows how the slope of the simulated cooling power behaves for different values of h_c. Calculating the slope of the experimental cooling powers leads to h_c values of 11.6 and 11.2 W m^{-2} K^{-1} for the thin and thick PDMS samples, respectively. Related to Figure 3 and Cooling Power Characterization.

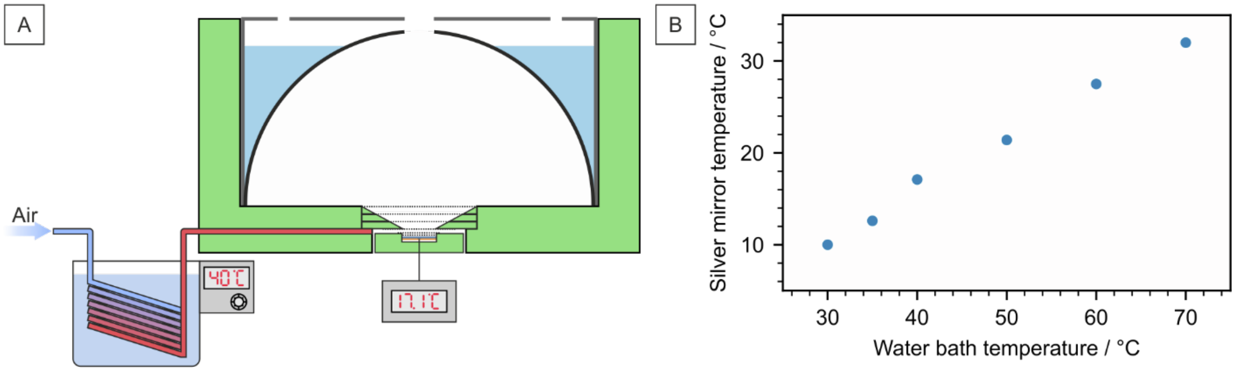

Figure S8. Tunable ambient temperature of the indoor setup. (A) Schematic of a part of the indoor setup. (B) Ambient temperature of the indoor setup as a function of water bath temperature. Related to Figure 4 and Variation of Environmental Parameters.

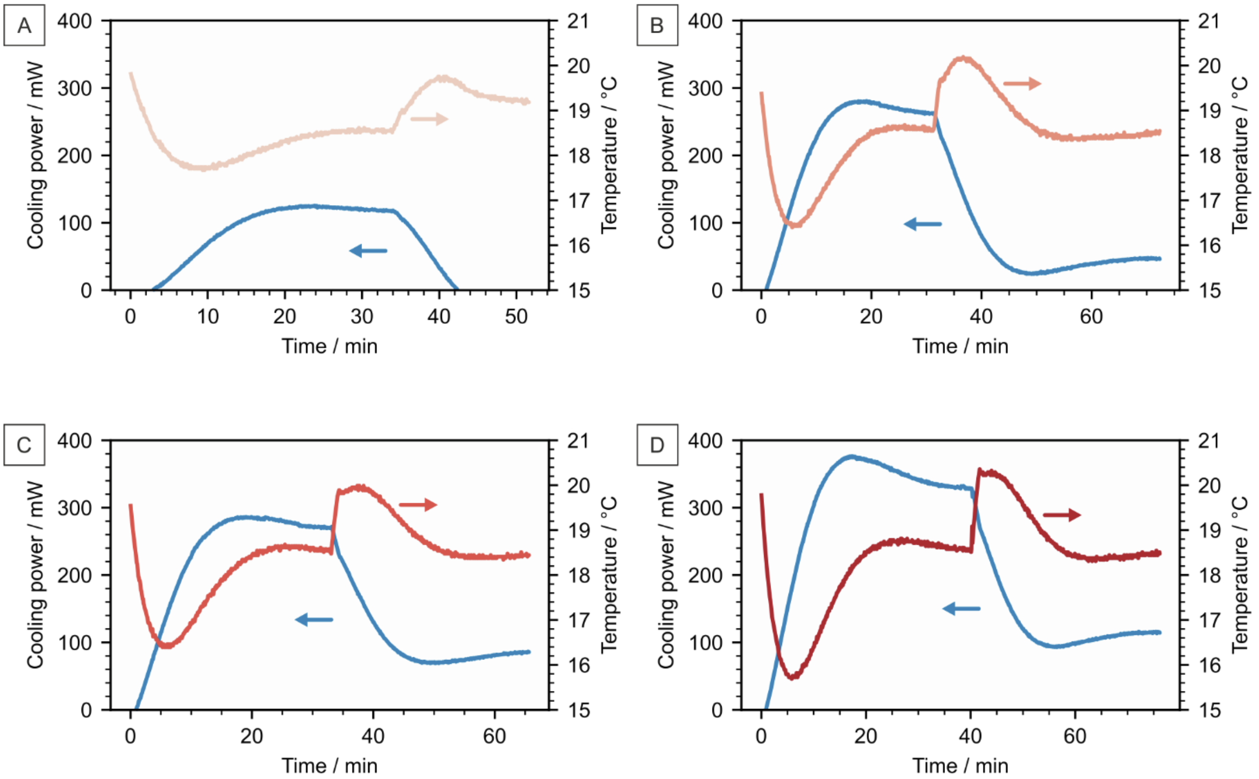

Figure S9. Cooling power measurements with and without a MIR filter for PDMS samples with a thickness of (A) 712 nm, (B) 8.6 µm, (C) 13.1 µm, and (D) 88.4 µm. The MIR filter is inserted into the indoor setup after temperature and cooling power reach a steady state. The insertion of the MIR filter leads to a significant decrease in cooling power for all samples.

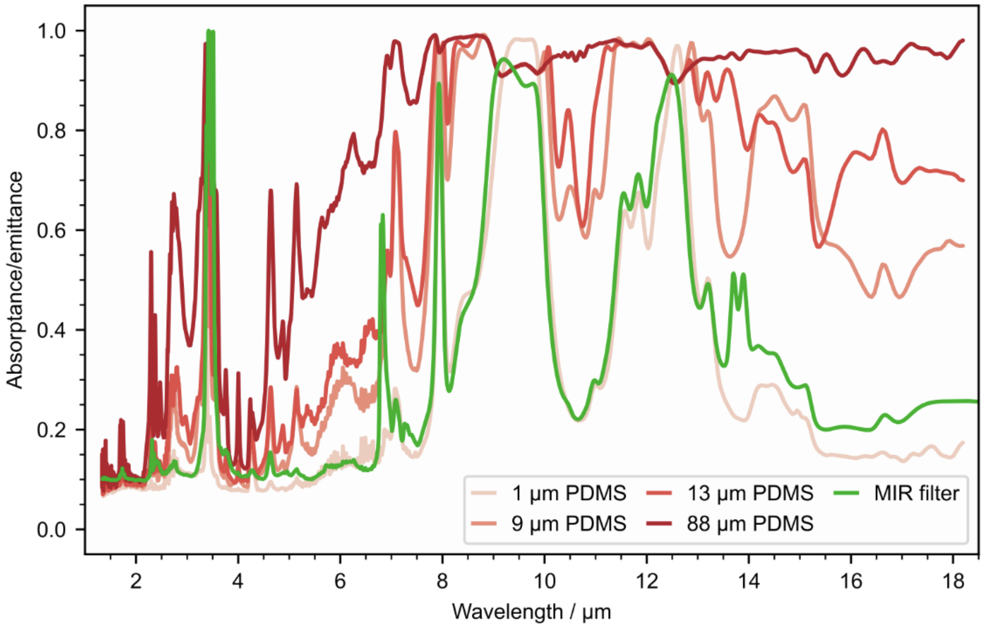

Figure S10. Absorption spectra for PDMS films of different thicknesses and the MIR filter. Decreasing thickness leads to higher emission selectivity.

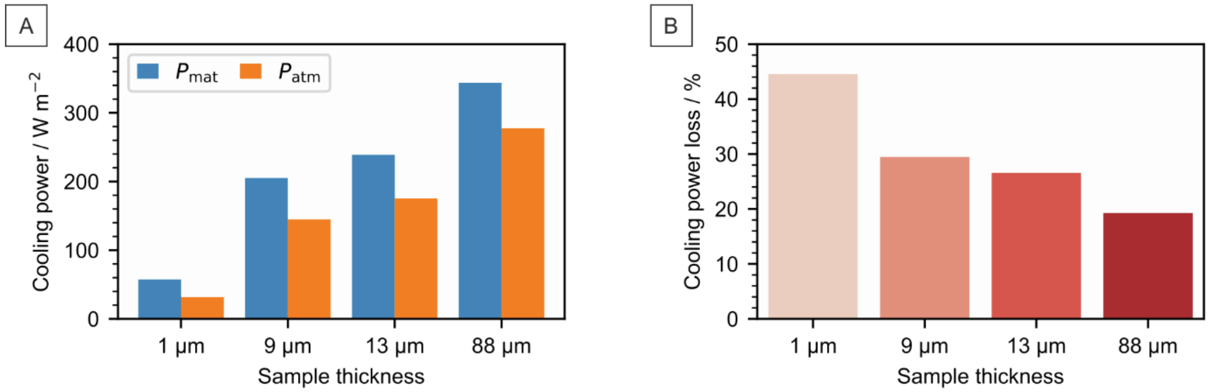

Figure S11. Simulation results for PDMS samples with different thicknesses in outdoor conditions. (A) Absolute contributions to the cooling power. (B) Cooling power loss. The loss is calculated as (P_{mat} – P_{atm}) / P_{mat}.
